# Rapid emotional processing in relation to trauma-related symptoms as revealed by magnetic source imaging

**DOI:** 10.1186/1471-244X-14-193

**Published:** 2014-07-05

**Authors:** Inga Schalinski, James Moran, Maggie Schauer, Thomas Elbert

**Affiliations:** 1 Department of Psychology, University of Konstanz, P.O. Box 905, 78457 Konstanz, Germany

**Keywords:** PTSD, MEG, IAPS, Shutdown, Dissociation, Depression

## Abstract

**Background:**

Traumatic stress leads to functional reorganization in the brain and may trigger an alarm response. However, when the traumatic event produces severe helplessness, the predominant peri-traumatic response may instead be marked by a dissociative shutdown reaction. The neural correlates of this dissociative shutdown were investigated by presenting rapidly presented affective pictures to female participants with posttraumatic stress disorder (PTSD), and comparing responses to a Non-PTSD control group.

**Methods:**

Event-related-magnetic-fields were recorded during rapid visual serial presentation of emotionally arousing stimuli (unpleasant or pleasant), which alternated with pictures with low affective content (neutral). Neural sources, based on the L2-surface-minimum-norm, correlated with the severity of the symptom clusters: PTSD, depression and shutdown dissociation.

**Results:**

For the early cortical response (60 to 110 ms), dissociation and PTSD symptom severity show similar spatial distributions of correlates for unpleasant stimuli. Cortical networks that could be involved in the relationships seem to be widespread.

**Conclusion:**

We conclude that shutdown dissociation, PTSD and depression all have distinct effects on early processing of emotional stimuli.

## Background

Repeated exposure to traumatic stress not only produces the core symptoms of posttraumatic stress disorder (PTSD), but can also produce symptoms of depression, dissociation and affective dysregulation. Dissociative symptoms that arise after trauma are common in patients who have been exposed to severe and repeated traumatic stress, particularly interpersonal trauma [1]. Persistence of dissociative responding seems to promote the development and maintenance of PTSD [2-4], and is associated with a greater severity of PTSD [5-7], and depression symptoms [8]. The relationship between dissociation and trauma is of considerable clinical and nosological significance, however the theoretical concepts have remained a source of controversy [9]. In the present study, trauma-related dissociation is understood as a biological response to an overwhelming stressor, in which the survivor finds themselves in a completely helpless condition. In such situations, the body responds to stress with a shutdown of perceptual, cognitive and affective information processing (for a detailed description of this concept see [10]). From an evolutionary perspective, shutdown dissociation might be seen as an adaptive response to life-threatening conditions during which neither flight nor fight is a viable options for survival: since the organism cannot defend itself or escape, it can only minimize harm, through immobility and functional sensory deafferentiation [11]. The shutdown of sensory and functional systems may later reoccur in response to trauma-related cues, intrusions or minor stressors [10]. Dissociative responding acts as a cognitive avoidance mechanism and reduces the awareness of aversive emotions such as extreme fear as well as overwhelming autonomic arousal [12]. In the context of exposure therapy, where therapeutic outcomes are attained by intensive reliving of the experience so that physiological fear responses can be habituated, and events integrated into memory, dissociation can become a severe obstacle.

Dissociative responding consists of a major distortion of attention through a perceptual shutdown of information processing. At the level of functional neural activity, this could mediated by cortico-subcortical top down processes. Abnormal cortical activity has been examined in several neuroimaging studies of PTSD, either measuring brain activity in a resting state or with stimuli designed to trigger trauma specific processing. The latter includes script driven imagery [13] or exposure to emotionally evocative pictures [14-19]. Functional neuroimaging studies using trauma scripts as stimuli suggest that there may be distinct neural circuitry involving frontal and limbic systems that distinguish between PTSD patients with and without dissociative responding. One study dividing PTSD patients into such groups: a non-dissociative PTSD group, with strong intrusions, and intense hyperarousal symptoms; and a dissociation group, showed correspondingly different patterns of functional neural activity. In comparing both groups to a Non-PTSD control, the PTSD patients with more hyperarousal symptoms also displayed lower bilateral medial frontal activity and left anterior cingulate activity, whereas the subgroup of patients with dissociative PTSD had increased right medial frontal, right medial prefrontal, right anterior cingulate activity. The higher prefrontal activity seems to co-occur with reduced amygdala activity in dissociative PTSD [13]. These results were interpreted as emotional over-modulation in dissociative PTSD, whereas an emotional under-modulation mediated by less intensive prefrontal inhibition of the limbic system was found in non-dissociative PTSD. Using a dimensional approach, Hopper and coworkers [20] found that the strength of dissociation was positively associated with the activity in the left medial prefrontal and right superior temporal cortices, and negatively correlated with the left superior temporal cortex. In a resting state study, Ray and colleagues [21] studied abnormal slow wave activity in the delta range (1.5 – 4 Hz) of the cortical magnetoencephalogram (MEG) in PTSD patients with torture exposure. Abnormal slow wave activity reflects structural and functional abnormalities in neural networks. They found an association between slow wave activity generated in the left ventrolateral frontal cortex and degree of dissociative responding. During recordings of the brain’s magnetic fields emotionally evocative photos have been used to assess the visual emotional processing [14,15]. Results indicate a deviant rapid network activity in the right frontal cortex in response to threatening stimuli. This response was followed by a less pronounced response in the parieto-occipital cortex. This cortical network pattern was interpreted as a vigilance-avoidance response, where the PTSD group showed an increased cortical response to salient stimuli followed by disturbed emotional modulation.

One important paradigm for examining the visual emotional processing is the rapid serial visual presentation (RSVP) of pictures from the International Affective Pictures System (IAPS). Elbert and co-workers [16] found evidence for an early change of aversive processing from the primary visual cortex to fronto-temporal regions and the amygdala in traumatized individuals compared to a Non-PTSD control group. Methodological studies of emotionally salient processing of visual stimuli at rapid presentation rates (3 to 5 Hz) revealed an effect of affective arousal at about 150 ms after the stimulus onset [17]. This effect reflects a very early differentiation between emotionally salient stimuli at initial stages of visual processing. This early posterior negativity (EPN) has been investigated in a wide variety of contexts e.g., [22,23]. The motivational system shows enhanced early selective processing of emotionally salient stimuli, which has the function of prioritizing the encoding of stimuli related to both sustenance (appetitive) and survival (defensive) of the organism. Thus, this early initiation in the face of threatening stimuli can be critical to survival [24,25]. Patients with PTSD show a hypersensitized level of processing, discriminating very early (60 to 110 ms) between threatening and non-threatening cues in cortical response [16], but show less affective discrimination in later processing [14].

In sum, neuroimaging studies suggest different brain responses in dissociative versus non-dissociative PTSD, which could either be dimensional or represent a distinct categorical pattern of a subgroup of patients with trauma-related illness. The RSVP paradigm has shown robustly different early visual processing in PTSD versus Non-PTSD controls, and at the behavioural level, that it was sufficient to activate the fear-network so that patients showed intrusive memories and flashbacks [16,17,26]. However, thus far there has been no study examining potential differences within the PTSD group, with varying levels of dissociation. Is the chronic hypersensitivity of early visual processing uniform across PTSD patients, or moderated by levels of dissociation or depression? Because of the inherent associations between trauma-related dissociation and psychopathology, particularly PTSD and depression, we decided to examine the respective symptom clusters separately as potential modulators of affective processing. We used event-related magnetic field potentials to investigate the following questions in a dimensional approach within the PTSD group (1) Is there abnormal emotional processing, indicated by different valence and arousal ratings? (2) Is it possible to reactivate the shutdown dissociation during the passive viewing task of emotionally salient photographs (when the fear-network is active)? (3) Does the heart rate, as an index of autonomic arousal during the presentation of unpleasant visual stimuli, differ from the heart rate while pleasant/ neutral stimuli are presented? (4) Are shutdown dissociation, PTSD and/or depression symptom severity associated with altered visual processing of arousing and neutral stimuli within the PTSD group, and how do these inherently associated psychopathologies converge at the level of brain activity during RSVP?

## Methods

### Structured clinical interview

Female participants were recruited for the study at the University of Konstanz outpatient clinic for refugees in Germany. They were referred to the clinic by human rights organizations, medical doctors or lawyers, for diagnostic assessment. Expert-psychologists carried out structured clinical interviews with the support of trained translators at least one week prior to the MEG. We conducted 49 interviews between April 2010 and February 2012 (inclusion criteria were female refugee with multiple traumatic experiences). Thirty-seven of the interviewed women were included in the PTSD group. Four subjects did not participate for the following reasons: two for technical reasons, one subject was too afraid of the laboratory, and one subject gave informed consent but showed severe dissociation at the beginning of the investigation so the procedure was aborted. Thirty-three participants who presented with PTSD symptoms were analysed in the present study. These were compared to 17 Non-PTSD controls with similar ethnic backgrounds, who were recruited from the general community and from the University of Konstanz. Participants in the Non-PTSD control group and most of the patients had been examined with the Cardiac Defense Paradigm [27]. Each participant was interviewed after providing informed consent. First, demographic data as well as medication were assessed (compare Table 1). Following this, the number of traumatic experiences was assessed using the sum of the event checklist of the Clinician Administered PTSD Scale [28]. For traumatic events, we made a distinction between the number of various traumatic event types that were self-experienced and the number of different traumatic event types that were witnessed. A traumatic event type was judged as self-experienced if the participant was the victim; or as witnessed if the participant had observed the traumatic event while someone else was threatened. For the PTSD diagnosis, we used the Clinician Administered PTSD Scale and summed its score for the symptom severity. Current comorbid disorders according to the criteria of the forth version of the Diagnostic and Statistical Manual of Mental Disorder (DSM-IV; such as depression, dysthymia, abuse or dependency of alcohol/illegal substances, suicidality and psychotic disorders) were assessed with the MINI International Neuropsychiatric Interviews [29]. The score on the Hamilton Rating Scale for Depression [30] estimated the degree of depression. To assess dissociative responding, we used the 13-item Shutdown Dissociation Scale (Schalinski I, Schauer M, Elbert T: The Shutdown Dissociation Scale (Shut-D), submitted.), developed by our research group based upon our concept of shutdown dissociation [10].

**Table 1:** Group means, standard deviations and differences of the demographic and clinical data for the PTSD and the Non-PTSD Group

	**PTSD**		**Non-PTSD**		**Statistics for group differences**	
	***M/n (range)***	***SD/%***	***M/n***	***SD/%***		
Age (Years)	36.74	9.67	31.88	6.83	*t*(49) = 1.85	*p* = .071
Education (Years)	5.59	5.46	18.68	3.46	*t*(46.05) = -10.41	*p* < .001
Regions of Origin N%					*χ*^*2*^(3, 51) = .11	*p* = .991
Middle and Far East	18	52.9%	9	53%		
The Balkans	5	14.7%	2	11.8%		
Africa	9	26.5%	5	29.4%		
India	2	5.9%	1	5.9%		
Number of Traumatic Event Types (Self-experienced)	5.47	2.35	1.24	1.40	*t*(47.41) = 8.05	*p* < .001
	(2-11)					
Number of Traumatic Event Types (Witnessed)	3.47	2.34	1.24	1.15	*t*(49.00) = 4.58	*p* < .001
	(0-9)					
Time elapsed since the worst traumatic event (Years)	9.74	8.82	7.45	4.80	*t*(32.11) = -1.09	*p* = .284
PTSD Symptom Severity	82.56	15.54	0.94	2.66	*t*(36.71) = 29.76	*p* < .001
	(55-113)					
Hamilton-Depression Severity	20.32	8.77	2.24	2.51	*t*(42.37) = 11.14	*p* < .001
	(6-37)					
Shutdown Dissociation Score	17.50	8.33	0.94	1.25	*t*(35.88) = 11.34	*p* < .001
	(2-33)					

*Note*. Mean (*M*), Standard Deviation (*SD*), absolute number of respondents (*n*), PTSD = Posttraumatic Stress Disorder. For group differences, *t*-tests were used for continuous variables and *χ*^*2*^ tests were applied for nominal variables.

### Demographical and clinical data

In the PTSD group, the region of origin was 52.9% Middle or Far East, 14.7% the Balkans, 26.5% Africa and 5.9% India. The Non-PTSD control groups’ origin was 53% Middle or Far East, 11.8% the Balkans, 29.4% Africa and 5.9% India. The ethnic composition of both groups was not statistically different; *χ*^*2*^(3, 51) = .11, *p* = .991. The age of the PTSD group ranged from 17 to 56 years (*M* = 36.7, *SD* = 9.7). The Non-PTSD group were on average *M* = 31.9 years old (*SD* = 6.8) and the age ranged from 22 to 49 years. There was no statistically significant difference in age between groups. On average, the participants in the PTSD sample were less educated when compared to the Non-PTSD control group (*t*(46.05) = -10.41, *p* < .001). On average the participants in the PTSD group had experienced *M* = 5.5 (*SD* = 2.4; range 2 to 11) and witnessed *M* = 3.5 different types of traumatic stressors (*SD* = 2.3, range 0 to 9). The following self-experienced events were reported most frequently: physical assault (79%), assault with a weapon (65%) and sexual assault with penetration (53%). Almost half of the PTSD sample (47%) was exposed to traumatic experiences in war-zones. The majority witnessed physical assaults (71%), 44% witnessed homicide and 38% witnessed a serious traffic accident. The Non-PTSD group was significantly less exposed to traumatic event types that were self-experienced *t*(47.41) = 8.05, *p* < .001 and witnessed *t*(49) = 4.58, *p* < .001). They had experienced on average *M* = 1.2 traumatic event types (*SD* = 1.4; range 0 to 4) and witnessed on average *M* = 1.2 traumatic event types (*SD* = 1.2; range 0 to 3). All participants in the PTSD sample fulfilled the DSM-IV criteria for PTSD. Additionally, all respondents with PTSD met the DSM-IV criteria for depression. None of respondents in the Non-PTSD group met the criteria for either PTSD or depressive disorders. None of the study participants fulfilled the criteria for current or past psychotic disorder or alcohol or substance abuse/ dependency. The PTSD group had significantly higher scores on the Clinician Administered PTSD Scale, Hamilton Depression Rating Scale and the Shutdown dissociation Scale compared to the Non-PTSD group (all *p* < .001). A part of the PTSD sample (*n* = 5 (15%)) was treated with psychoactive medication. Five patients in the PTSD group were taking antidepressants (*n* = 5 (15%)) and one of these was also taking neuroleptic medication (*n* = 1 (3%)). None of the participants in the Non-PTSD group were taking psychoactive medication at the time of testing. None of the participants took medication targeting the cardiovascular system such as digitalis, beta-blockers or anticholinergics. All participants reported normal vision (with correction). Further, all participants wrote with their right hand. The handedness score (100 = 100% right-handed) was on average *M* = 95.7 (*SD* = 14) in the PTSD group and *M* = 96.5 (*SD* = 6.1) in the Non-PTSD group.

### MEG apparatus and physiological assessment

The magnetic fields were measured with a 148-channel whole head magnetometer (MAGNES 2500 WH, 4D Neuroimages, San Diego, USA) with a sampling rate of 678.17 Hz. Data were recorded continuously with a band pass filter between 0.1 and 200 Hz. Electrooculogram (EOG) and electrocardiogram (ECG) were measured with a SynAmps amplifier (NEUROSCAN Laboratories, Sterling, VA, USA). Four Ag/AgCl electrodes were attached (two near the left and right outer canthus and two above and below the right eye) to obtain the vertical and horizontal EOG. An electrode attached above the right zygomatic bone provided grounding. To record the ECG, two Ag/AgCl electrodes were positioned above the right collarbone and the left lower costal arch. The R wave of the heart rate was semi-automatically detected using BESATM software (module: create triggers) and the R-R-intervals were converted to heart rate. The heart rate was assessed within each of the blocks and averaged across the pleasant/neutral and unpleasant/neutral blocks (compare below: Experimental Design).

### Stimuli

Based on normative valence and arousal ratings, 100 arousing unpleasant (e.g., mutilations, assaults, etc.), 100 pleasant (e.g., wedding, children, etc.), and 100 neutral (e.g., neutral faces, daily life scenes, etc.) colour photographs were chosen from the IAPS [31]. The three categories differed significantly from each other in IAPS normative valence ratings. Arousal ratings did not differ for pleasant and unpleasant contents, but the mean arousal rating was different for neutral contents. Brightness, contrast, and colour spectra of the stimuli did not differ across the picture categories. The complexity of the stimuli was rated prior to the testing and did not differ between the three categories.

### Experimental design

The stimuli were presented in 6 blocks with three repetitions of each block. One type of block (unpleasant/neutral block) consisted of 100 unpleasant and 100 neutral pictures from the IAPS. The other type of block (pleasant/ neutral) contained 100 pleasant and 100 neutral pictures. The neutral pictures were identical in both blocks. The sequence of blocks was presented in an alternating order and the sequence was balanced so that half of the participants started with the unpleasant/neutral block and the other half with the pleasant/ neutral block. The duration of one block was 66.67 s, and the frequency of picture presentation was 3 Hz, without any interval between the stimuli. The time between two blocks was set between 5 s and 10 s. The order of pictures within the block was pseudo-randomized to keep the probability of the picture alternation sequence constant. Therefore the order in which the pictures were presented within the block was determined so that the chances of an unpleasant picture following a neutral picture were exactly 50% and vice versa. The summed average of prior stimuli will consist of 50% neutral pictures and 50% unpleasant pictures in the unpleasant/ neutral blocks and 50% neutral with 50% positive for the pleasant/ neutral blocks. This was applied for two reasons, firstly to avoid any noticeable pattern in the series, which might influence participant reactions. Secondly, to ensure that our baseline measures, which are derived from 100 ms before a given stimulus onset, is not biased.

### Testing procedure

The University of Konstanz’s ethics committee approved the protocol. Participants were paid €20 and travel costs. Prior to the testing, the participants were informed in detail about the procedure and were familiarized with the laboratory setting where recording would take place. After this, the participants provided informed consent. One under-aged participant together with her legal representatives also signed the form. The Edinburgh Inventory was used to assess the handedness [32]. Participants were then seated in a magnetically shielded chamber and their head shapes were digitized with a Polhemus 3 Space Fasttrack (Polhemus, Colchester, VT, USA). Five index points (left and right periauricular points, nasion, pseudo-Cz and pseudo-inion point at the forehead) were determined to calculate the relative head position within the MEG helmet for source analysis. Finally, the participant was placed in a supine position and the head was positioned under the MEG sensors. The data were recorded in supine position. The participants were instructed to keep their eyes focused on a cross that appeared in the middle of the visual stimuli. They were further instructed to avoid any eye movements, to minimize blinking during RSVP, and avoid any other physical movement. After the MEG data were collected, a psychologist interviewed the participants about their experiences and clinical symptoms during the MEG data collection. Intrusive memories during the data collection were assessed. Participants were asked about visual, acoustic, haptic, as well as odour recollections of the traumatic memories. Further, any upsetting feelings were documented. After the data collection, the tendency towards shutdown dissociation was rated qualitatively ranging from 0 (not at all), 1 (a little bit), 2 (moderately), 3 (strongly) to 4 (very strongly) for the period of picture presentation, using the 13-item Shutdown Dissociation Scale (Schalinski I, Schauer M, Elbert T: The Shutdown Dissociation Scale (Shut-D), submitted.*)*. Sum scores could range between 0 and 52. After the interview, participants did a computer-based valence and arousal rating. A subset (75 pictures; 25 from each category) of the 300 stimuli was presented for 6 s on a 15-inch screen. This was followed by the manikin scales [33], which subsequently appeared below the pictures. These scales measured valence followed by arousal.

### Data analysis

The MEG data were first corrected for heartbeat-related artefacts using 4D Neuroimaging cardiac-remover-software. For time segments with R-wave artefacts, an average MEG was subtracted, determined as a moving average over 20 heartbeats. Thereafter, noise reduction for external disturbances was achieved through distant reference sensors. For further pre-processing and analysis, data were transferred to BESA® software (MEGIS Software GmbH, Munich, Germany; Version 5.3). The data was manually scanned for epochs containing eye-blinks, which were deleted from further analysis. Prior to averaging, a forward high pass filter of 1 Hz was set (6 dB/ octave). The artefact scan tool from BESA® software was applied for additional artefact rejection. The threshold for the amplitude was set to 2500 fT and the gradient threshold was set to 500 fT/sample. Bad channels were interpolated where possible or rejected from further analysis. There were never more than 5 bad channels in total (out of 148, < 5%) for any of the participants. Our baseline was set at 100 ms prior to stimulus onset. In our laboratory the lag between the trigger and the stimulus occurrence was 20 ms and was taken into account for averaging. After averaging, a zero-phase low pass filter with a cut-off frequency of 40 Hz was applied with a slope of 24 db/ octave. The number of trials in the PTSD group was on average *M* = 257 (*SD* = 35). In the Non-PTSD on average of *M* = 276 (*SD* = 18) remained for averaging.

### Magnetic source imaging: L2- minimum-norm estimate in source domain

The minimum-norm estimates were calculated with BESA. The spherical head model was used and sensor amplitude data were used to calculate the estimates. The transformation was achieved with an L2 Minimum-norm-pseudoinverse calculation. The technique estimates the source activity without a priori assumptions about the sources’ location and activity or the number of sources. The inverse problem is addressed by generating dipole solutions of the sensor data with the smallest amount of power for all dipole sources at each time point. Hämäläinen and Ilmoniemi [34] hypothesized that the L2 minimum-norm provides the most parsimonious and therefore most realistic approximation of real brain activity. To attain the minimum-norm estimates, first the forward solution (leadfield matrix) of all sources data is calculated in the spherical head model (azimuthal and polar direction). Further, the source activities are computed from the sensor data with the help of an inverse regularized estimation of the noise covariance matrix of the sensor data. Tikhonov regulation constant was set to 0.1 and applied to invert the calculation. In order to compensate for the tendency of the minimum-norm solution to favour superficial sources, spatial depth-weighting method was also used. Depth weighting for the mean norm of the recursive leadfields was applied using subspace correlation after single source scan ρ^2^. The data with 15% lowest global field power are selected for noise estimation. The source activity of each regional location is estimated as the root mean square of the sources’ components. The source activity of evenly distributed regional sources is computed at 10% and 30% below the standard brain surface. The larger of the two corresponding source activities were used for further calculation. The volume grid size for imaging was set to 12 mm.

### Selection of time window of interest

The main goal of the present analysis was to assess responses to the RSVP paradigm in the following dimensional scales: shutdown dissociation (specifically that assessed during the RSVP presentation, as opposed to over the last 6 months); PTSD; and depression symptom severity in visual processing of affective material. Therefore correlations of the global field power of the minimum-norm estimate were calculated and considered as important when at least 8 consecutive data-points (11.8 ms or longer) were significantly correlated. The correlations were assessed within the PTSD group to avoid global correlations due to the Non-PTSD group.

### Statistical analysis

Analyses were performed using R version 2.15.1 and SPSS 20.0; alpha level was set at .05. To compare the demographic and clinical data between the PTSD and Non-PTSD group, *t*-tests were used for continuous dependent variables and *χ*^*2*^ tests were applied for nominal variables (using SPSS). Correlations and *t*-tests of the minimum-norm data were calculated with R. Rosenthal’s *r* was calculated as effect size [35].

## Results

### Behavioural responses

**Figure 1:**
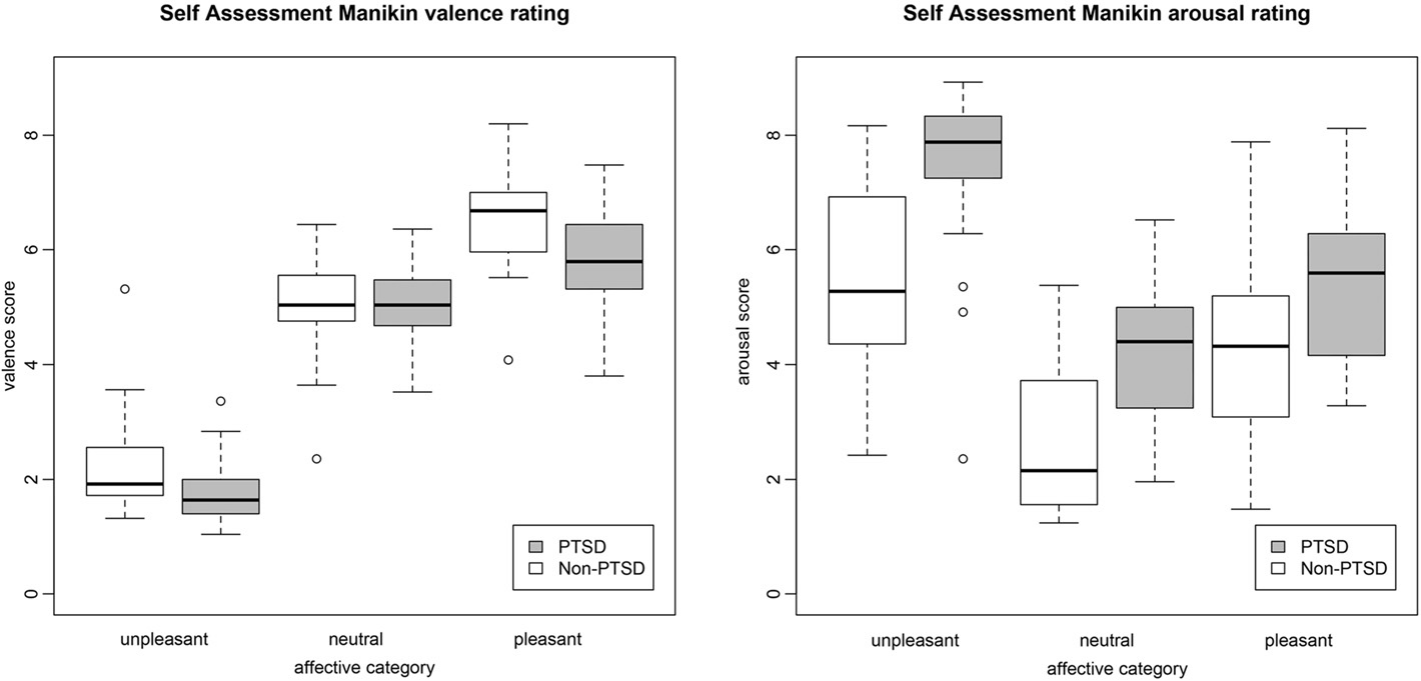
**This figure presents the Self Assessment Manikin valence and arousal ratings of the PTSD and Non-PTSD group as a function of the picture categories (unpleasant, neutral and pleasant).** The box frames the lower and upper quartile. The bar inside the box shows the median and error bars indicate the variability outside the lower and upper quartiles. Circles indicate outliers. PTSD = Posttraumatic Stress Disorder.

Figure 1 presents the boxplot of the valence and the arousal rating separately for the PTSD and Non-PTSD group. Valence and arousal rating were analysed with in separate repeated ANOVAs. Mauchly’s test indicated that the assumption of sphericity had been violated for the main effect of valence, *χ*^*2*^(2) = 11.94, *p* = .003. Therefore degrees of freedom were corrected using Greenhouse- Geisser estimates of sphericity (*ϵ* = .82 for the main effect of valence). As expected, the valence ratings differed across the affective category, *F*(1.63, 78.41) = 451.83, *p* < .001, *η*^*2*^ = .90. In addition, the analyses yielded evidence for interactive effects between the group and the valence rating (*F*(1.63, 78.41) = 3.50, *p* = .044, *η*^*2*^ = .07). Additionally, the group effect was significant *F*(1, 48) = 4.44, *p* = .040, *η*^*2*^ = .09.

Arousal ratings for all participants varied across the picture categories, with unpleasant and pleasant pictures rated as more arousing than neutral pictures. Further, unpleasant pictures were rated as more arousing compared to pleasant pictures. The analysis revealed a significant main effect of affective category: *F*(2, 96) = 113.11, *p* < .001, *η*^*2*^ = .70. Furthermore, there was evidence for an interactive effect (*F*(2, 96) = 4.26, *p* = .017, *η*^*2*^ = .08). The analysis yield a main group effect (*F*(1, 48) = 19.32, *p* < .001, *η*^*2*^ = .29).

### Clinical symptoms during the RSVP

Immediately after the MEG examination, a psychologist interviewed the participant regarding intrusive memories and the strength of shutdown dissociation during the testing. All patients reported upsetting feelings such as fear, anxiety or sadness during the testing. More than half of the PTSD patients (*n* = 20) reported intrusive visual memories triggered by the RSVP. Further, ten patients with PTSD recollected haptic and acoustic intrusions. An odour perception was reported by 10% (*n* = 5) of the cases. None of the participants in the Non-PTSD control group reported any such recollections.

### Relation between clinical symptoms and responding

Correlations were assessed within the PTSD group. Patients with stronger PTSD symptoms also reported greater shutdown dissociation in response to the RSVP (*r* = .45, *p* = .009). Moreover, shutdown dissociation in response to RSVP was also correlated with depression symptoms (*r* = .42, *p* = .016). Further, PTSD symptom severity was associated with depression severity (*r* = .52, *p* = .002).

### Heart rate response

Heart rate was recorded during exposure to the blocks (unpleasant/ neutral and pleasant/ neutral). The PTSD patients had on average a heart rate of *M*_*unpleasant/neutral*_ = 68.2 (*SD* = 6.4) beats per minute (bpm), in the unpleasant/neutral and *M*_*pleasant/neutral*_ = 67 (*SD* = 6.7) bpm in the pleasant/neutral block. The Non-PTSD group had on average heart rates of *M*_*unpleasant/neutral*_ = 66.3 (*SD* = 7.6) bpm in the unpleasant/neutral block and *M*_*pleasant/neutral*_ = 65.5 (*SD* = 8) bpm in the pleasant/neutral block. An analysis of variance with one repeated factor of block (heart rate in the unpleasant/ neutral block versus pleasant/neutral block) and group (PTSD versus Non-PTSD control) was performed. The analysis yielded a significant main effect of block; *F*(1, 47) = 12.92, *p* < .001, *η*^*2*^ = .22. There was no interaction between the factor and group *F*(1, 47) = 0.53, *p* = .469, *η*^*2*^ = .01, nor was the group effect significant *F*(1, 47) = 0.64, *p* = .427, *η*^*2*^ = .01. Further, correlates between the heart rate and symptom severities were assessed within the PTSD group. For the PTSD group, greater PTSD symptom severity was correlated with higher heart rates during both the pleasant (*r* = .38, *p* = .033) and unpleasant blocks (*r* = .38, *p* = .032), but no significant associations were observed between shutdown dissociation in response to the RSVP and the mean of the heart rate in either the pleasant (*r* = .02, *p* = .910) or unpleasant blocks (*r* = .01, *p* = .940).

### Minimum-norm estimates in source space

To validate the paradigm, the magnetic counterpart of the EPN was assessed by the average of the minimum-norm estimated voxel amplitude from 160 to 300 ms (including 94 data points). An effect of affective arousal was found for the pleasant vs. neutral condition in all participants: *t*(49) = 2.25, *p* = .029, *r* = .31; *M*_pleasant_ = 1.43, *SD*_pleasant_ = 0.36; *M*_neutral_ = 1.38, *SD*_neutral_ = 0.34. The EPN counterpart of the unpleasant/ neutral block was also significant: *t*(49) = 2.82, *p* = .007, *r* = .37; (*M*_unpleasant_ = 1.41, *SD*_unpleasant_ = 0.32; *M*_neutral_ = 1.36, *SD*_neutral_ = 0.32).

### Time windows of interest and group comparison

**Figure 2:**
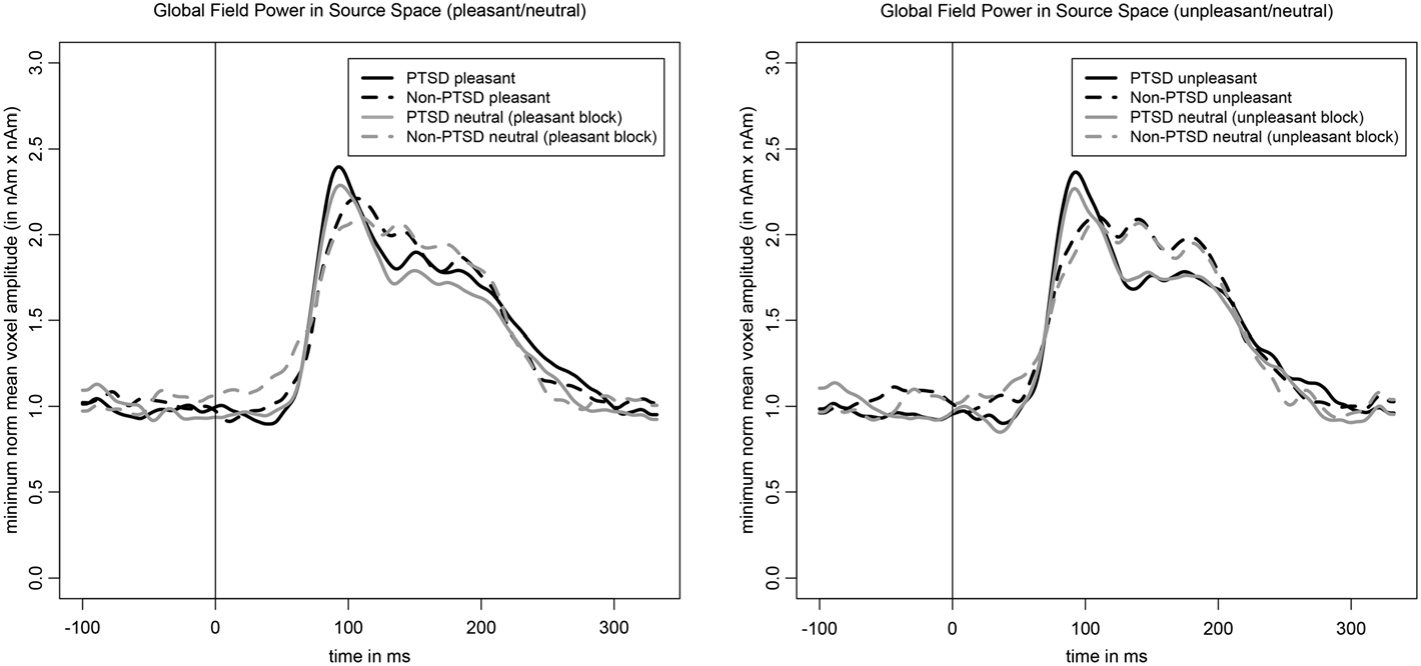
**This figure presents the global field power in source space averaged separately for the PTSD (solid lines) and Non-PTSD (dashed lines) group and separately for the picture category (black lines = high arousing emotional stimuli; grey lines = neutral stimuli).** Stimulus onset is the time point 0, indicated by the vertical line. PTSD = Posttraumatic Stress Disorder.

Figure 2 shows the global field power in source space. First we examined the correlations between the global field power in source space and the psychological variables of shutdown dissociation, PTSD and depression, across the different levels of the RSVP (unpleasant/neutral and pleasant/neutral pictures). Due to significant correlations between 60 to 110 ms after the stimulus presentation for shutdown dissociation, PTSD and depression symptom severity, the time window of interest was determined from 60 to 110 ms. Depression severity was associated with a time-window from 228 to 245 ms. Arousing visual stimuli (unpleasant and pleasant) compared to the respective low arousing neutral pictures produced stronger minimum-norm estimates in the PTSD group for the time window 60 to 110 ms; for the unpleasant/neutral block *t*(32) = 3.01, *p* = .005, *r* = 0.47, and for the pleasant/neutral block *t*(32) = 2.94, *p* = .006, *r* = 0.46. In contrast, the Non-PTSD control group showed this effect only for high arousing unpleasant versus neutral pictures; for the unpleasant/neutral block *t*(16) = 2.12, *p* = .050, *r* = 0.47, but not for the pleasant/neutral block *t*(16) = 1.22, *p* = .239, *r* = 0.29. For the time window 228 to 245 ms, there was neither a group difference nor a within group effect (all *p* > .050). There was a significant time window of a group difference (PTSD versus Non-PTSD control group) from 128 to 143 ms in the unpleasant condition (compare Additional file 1).

### Correlates for the time window of interest 60 to 110 ms within the PTSD group

**Figure 3:**
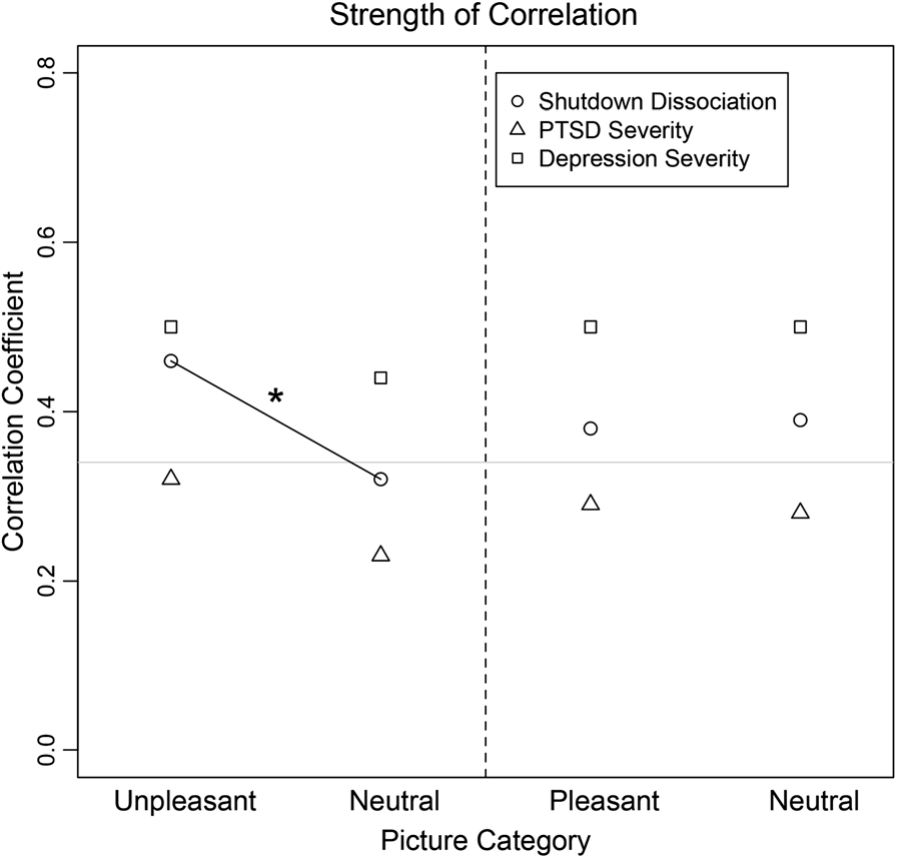
**This figure presents the Pearson correlation coefficient between the surface minimum-norm estimate in source space and the psychopathological scale (circle: Shutdown dissociation; triangle: Posttraumatic Stress Disorder (PTSD) symptom severity; rechtangle: Depression symptom severity) as a function of picture category.** The grey horizontal line presents the significance level indicating significant correlations above the line. The dashed line separate the blocks presenting the results of the unpleasant/neutral block at the left side and the results of the pleasant/neutral block at the right side. The black solid line and * indicate the significant difference between the correlation coefficient.

**Figure 4:**
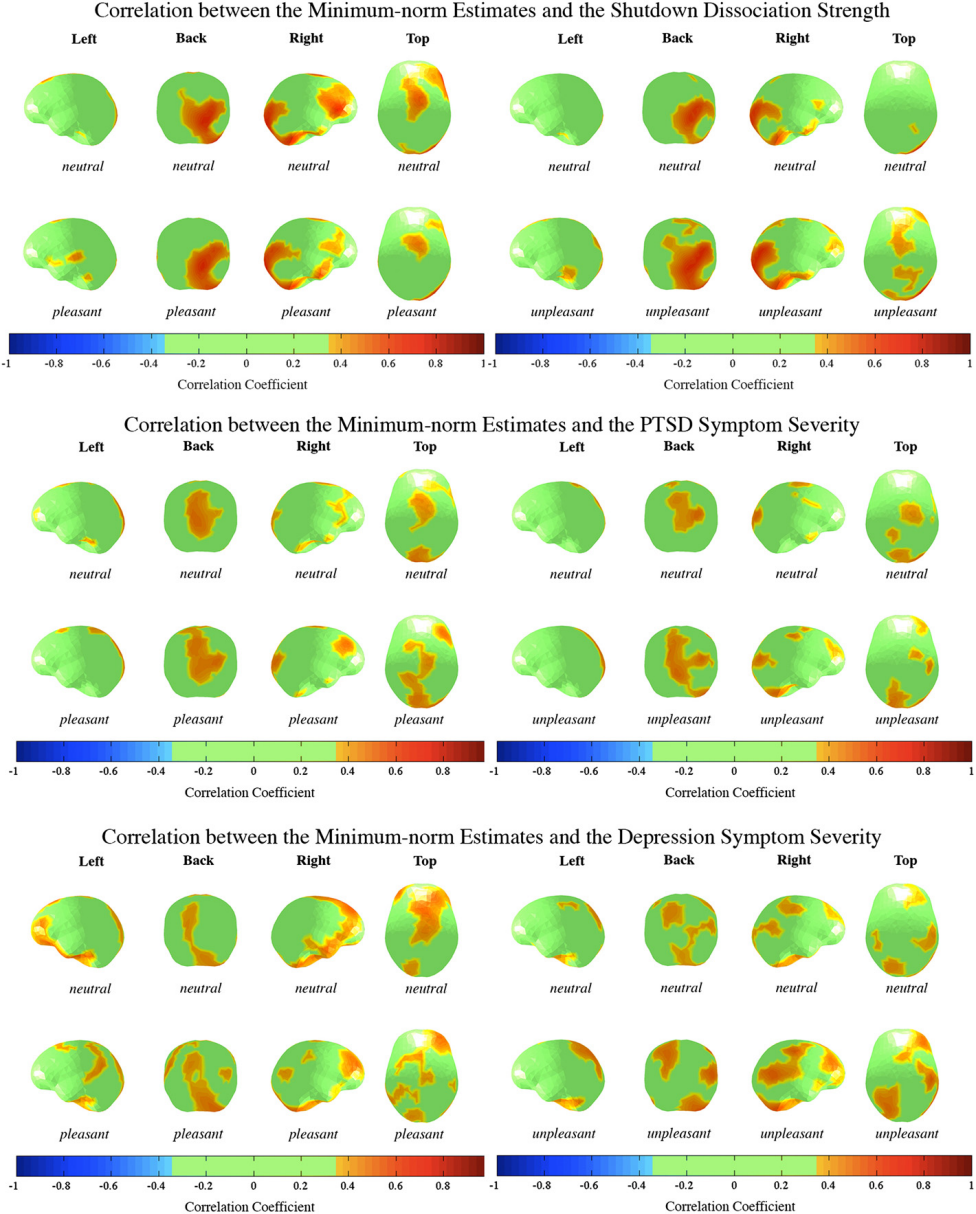
**Brain maps present the correlation between the minimum-norm estimate (average from 60 to 110 ms) and the shutdown dissociation strength in response to the processing of rapidly presented pictures, PTSD symptom severity and depression severity, projected on schematic cortical surfaces.** Brain maps are shown across the picture category and block type for the PTSD group. The brain maps are presented from different perspectives (left, back, right and top view). Correlations between -.3 and .3 were supressed. PTSD = Posttraumatic Stress Disorder.

The correlational strength of the average minimum norm estimates of the different conditions and the symptom severity (shutdown dissociation, PTSD and depression) are presented in Figure 3. Figure 4 shows significant correlations for each location of the minimum-norm estimate from 60 to 110 ms with the shutdown dissociation score, the PTSD symptom severity and the depression scores. Due to interrelation of the psychopathological scales, the common variance of the neural brain response was calculated and summarized in Table 2. The highest concordance of brain correlates (67%) was observed for the unpleasant picture category related to shutdown dissociation and PTSD symptom severity.

**Table 2:** **Similarities (r**^**2**^**) of brain correlates across conditions and symptom severities**

	**Shutdown dissociation/PTSD**	**Depression/shutdown dissociation**	**PTSD/depression**
Unpleasant	67%	31%	33%
Neutral (unpleasant/neutral block)	26%	26%	26%
Pleasant	42%	15%	28%
Neutral (pleasant/neutral block)	41%	25%	28%

*Note.* All *p* < .0001. Similarities are measured as proportion of variance explained, i.e. as squared correlation coefficient. PTSD = Posttraumatic Stress Disorder.

### Correlates for the time window of interest 228 to 245 ms within the PTSD group

For both affective conditions, the correlation with the minimum-norm estimation of the condition across 228 to 245 ms reached significance with the depression score (pleasant condition: *r* = .40; unpleasant condition: *r* = .37, *p* < .05), but not with the shutdown dissociation strength or PTSD symptom severity (all *p*s > .2). No significant associations were observed in the neutral condition of either block.

## Discussion

The present study examined processing of affective material in a sample of severely traumatized women with PTSD and varying degrees of dissociation compared to women without PTSD on multiple dimensions (behaviour, heart rate, subjective and neural processing). Using a dimensional approach, we assessed the early dynamics of visual processing in response to RSVP with respect to shutdown dissociation, PTSD and depression symptom severity.

Despite obvious cultural differences, the IAPS valence ratings of the present Non-PTSD sample are in accord with the normative ratings of the American sample. Arousal ratings were higher for unpleasant compared to pleasant pictures. On the behavioural level, PTSD patients showed different valence and arousal ratings. They rated unpleasant stimuli as more unpleasant compared to the Non-PTSD group. Further, they rated the arousal level of unpleasant and neutral stimuli as higher compared to the Non-PTSD control group. This finding of different ratings for unpleasant stimuli is in line with a previous study with PTSD patients showing exaggerated emotional responding [36]. PTSD patients show more extreme ratings (e.g. very negative, very arousing), making more of a distinct categorization rather than graded evaluation of the stimuli. The arousal rating differed between the pleasant and unpleasant stimuli in both PTSD and Non-PTSD controls, with unpleasant stimuli rated as more arousing. Consistent with the arousal ratings, the heart rate data confirmed a higher physiological arousal in the unpleasant/neutral block compared to the pleasant/neutral block for both groups. Although the unpleasant pictures were not personalized for the traumatic events, 60% of the PTSD sample experienced intrusive memories of their own trauma in response to the stimulation. The observation is consistent with the theory of a fear/trauma network, i.e., an interconnected network of neural representations formed through multiple threatening experiences. It encompasses sensory, cognitive, physiological, and emotional experiences and includes the action disposition related to the experience. When a few representations within this network (e.g. the sight of blood or weapons, cognitions, like “I cannot do anything”) become activated, the excitation will begin to spread through this interconnected excitatory network and activation of other trauma-related memory traces will have a low threshold see, e.g., [37]. The dominant peri-traumatic response – fear/anger or dissociative responding – will also be more likely to appear. Indeed, those PTSD patients who reported more intense shutdown dissociation in their daily life experienced more of these symptoms also during the emotional visual processing. To overcome the intrusive memories, the shutdown of perceptual channels, bodily functions and emotions may be rewarding in the short-term, as it interrupts the perception of trauma reminders and thus reduces the symptoms and autonomic arousal [10]. However, in the long-term this response will also disrupt psychosocial functioning and will leave the survivor without behavioural control and proper regulation of emotions [38].

### Minimum-norm estimates and brain correlates

Within the PTSD group, an arousal effect (mean of 60 to 110 ms) was found, showing higher global field power in source space in the pleasant and unpleasant compared to the neutral conditions. This arousal effect was also observed in the Non-PTSD control group in the unpleasant versus neutral condition, but not for the pleasant versus neutral condition. Elbert and co-workers [16] also found a very early arousal modulation in a sample of PTSD using the RSVP design. The affective modulation in the Non-PTSD sample could represent a higher behavioural importance of more arousing threatening stimuli compared to less arousing pleasant stimuli. The present study used a dimensional approach to assess the modulation of emotional processing. Shutdown dissociation, PTSD symptom severity and depression severity were all inherently associated with each other. The interrelations are described in literature: persistent trauma-related dissociation has been shown to be strongly associated with PTSD [2,3]. A person that went through a greater number of different types of traumatic events is more likely to develop PTSD and a co-morbid depressive disorder [39]. At the same time, the PTSD symptom severity is related to more severe dissociative responding [40]. So far, no studies have assessed how cumulative trauma affects the temporal pattern of the development of trauma-related symptoms, but it has been shown that dissociative symptoms play a role, even years after the traumatic experiences [41].

The analysis of the differential effects of the shutdown dissociation, PTSD and depression symptom severity addresses the issue of complex psychopathology in this sample and shed light upon the influences on emotional processing. Regardless of the picture category, positive correlations of the minimum-norm estimates with depression severity were found at a very early stage of visual processing between 60 and 110 ms. Shutdown dissociation was more strongly correlated with the processing of unpleasant/threatening stimuli compared to the correlates of the neutral condition. In contrast, for both levels of the pleasant/neutral block, positive associations between shutdown dissociation during testing and minimum-norm estimate were observed for the early time window. These very early effects of visual processing of salient emotional stimuli are consistent with previous findings [15,16]. The standard account of early processing holds that crude perceptual information can be relayed to important emotion centres of the brain before undergoing more sophisticated cortical processing [42,43]. Depression severity seems to affect the cortical processing regardless of the picture category. In contrast, the shutdown dissociation differentially modulates the processing of the unpleasant compared to neutral pictures. This is consistent with the theory of a fear-network, whereby shutdown dissociation is one possibility for the peri-traumatic response that in the course of multiple traumatic experiences may have become the primary mode of responding. From its brain activation pattern very early after stimulus onset, we might speculate that the shutdown dissociation enhances immediate and crude visual processing of threat cues. Cortical as well as subcortical networks that could be involved in the relationships are likely to be widespread (compare Figure 4). Shutdown dissociation and PTSD symptom severity show a 67% overlap of significant correlations for unpleasant stimuli, which is considerable but does not suggest that the two measures are identical. Although one could anticipate fundamental processing differences between a shutdown dissociation response and the hyperarousal that characterizes some PTSD patients, in terms of the peri-traumatic psychophysiological defence response, both scales show similar but not identical modulations of the brain circuits when the traumatized brain is confronted with unpleasant/threatening stimuli. Examining the results in further detail, the lower overlap between the brain’s correlate of shutdown dissociation and PTSD symptom severity for neutral stimuli suggest a qualitatively differential modulation of the processing for the two clinical syndromes. Additionally, the examination of the correlations with the minimum-norm estimates in the unpleasant condition and the standardized difference between PTSD symptom severity and shutdown dissociation (the residuals) result in non-significant correlations. The overlap may indicate that shutdown dissociation appears at the upper end of the posttraumatic stress response, rather than being uniformly present in all survivors with PTSD. DSM-5 includes, as a subtype, PTSD with prominent dissociative symptoms. Our data would argue for a dimensional/severity difference rather than a separate category, as in our sample, high shutdown dissociation is inherently associated with more severe PTSD. The high cortical overlap in the processing of threatening/unpleasant pictures raises the question of whether dissociation relates to a distinct categorical construct or appears as a modification of brain circuitry with increasing symptom severity. A greater exposure to traumatic stressors would increase PTSD symptom severity and also make peri-traumatic shutdown more likely, which then might replay as dissociative responding when cued by trauma-related reminders. A recent study found evidence of a dissociative symptom cluster that is correlated with the core PTSD symptoms and associated with higher PTSD symptom severity as well as more severe co-morbidity pattern [44]. It is likely that dissociation, with its ongoing disruption of integrative processes, would play a key role in the severity and maintenance of PTSD.

Depression symptom severity also correlates with a pattern of brain activity, however, there are clear differences in functional brain activity between depression and the other two symptom clusters. In contrast to the early effects of visual processing and depression severity, the later time window from 228 to 245 ms revealed only significant correlations with the minimum-norm estimates and the depression strength in the high arousing conditions (pleasant and unpleasant). These effects seem to be specific for the emotionally salient stimuli categories and present differential affective cortical processing. Usually, patients with depressive disorders show lower cortical modulation for affective arousal stimulation [45]. In contrast, the present results suggest that the depressive brain reacts more strongly towards arousing content than neutral content. This is in line with the results from another study that found the complementary modulation, namely hyperactivity to arousing stimuli in patients with depression and comorbid anxiety disorders [46,47]. Further, our results suggest that increasing severity of depression in a sample of patients with PTSD is associated with more pronounced response to highly arousing stimuli.

## Conclusion

Repeated exposure to traumatic stressors may result in PTSD, shutdown dissociation, and depression, whereby these three symptom clusters are interrelated but not interchangeable. All three are not only distinct clinical manifestations but also appear as different forms of functional brain organisation. Differential effects become visible in time (60-110 ms vs. 228-245 ms), space (correlations of regional pattern of activation with symptom severity) and affective arousal. With the dimensional approach, we could show that in an early time window, affective modulation of cortical response is associated with all three symptoms clusters, in an overlapping but also partly differential pattern. Regardless of the emotional salience, these variables seem to affect the streams of visual emotional processing. The stronger correlation between the shutdown dissociation and the minimum-norm estimate in the unpleasant versus neutral condition indicated differential processing. In the later time window (228 to 245 ms), selective arousal modulation associated with depressive symptoms could also be observed. The brain regions involved cause widespread activity. A high concordance of brain correlations was found for shutdown dissociation and PTSD severity in the unpleasant condition, but this overlap was much lower in the neutral condition. In sum, these results would support a model in which increasing exposure to traumatic stress, brain processing becomes altered on qualitatively different dimensions, as captured by symptoms of PTSD, depression and dissociation. That is, survivors of traumatic stressors present with a set of different functional reorganization of brain activity and hence a comparison of patients with and without PTSD may produce quite variable results if the other dimensions as well as their intensities are not considered.

## Competing interests

The authors declare that they have no competing interests.

## Authors’ contributions

MS & TE developed the study concept. TE and IS contributed to the study design. Data collection and data preprocessing were performed by JM and IS. IS developed scripts to analyse the event-related magnetic fields and performed the data analysis and interpretation under the supervision of TE. TE and IS drafted the paper, and JM and MS provided critical revisions. All authors approved the final version of the paper for submission.

## Pre-publication history

The pre-publication history for this paper can be accessed here:

http://www.biomedcentral.com/1471-244X/14/193/prepub

## Supplementary Material

Additional file 1 **Brain maps present the minimum-norm estimate (average from 128 to 143 ms) in the unpleasant condition for the Non-PTSD control group and the PTSD group.** The brain maps on the lower line show the significance of the group difference between the Non-PTSD control and PTSD group. The brain maps are presented from different perspectives (left, right, top and back view). Posttraumatic Stress Disorder. Click here for file
