# Supplementary figures and images for: Rapid emotional processing in relation to trauma-related symptoms as revealed by magnetic source imaging
Source: BMC Psychiatry. 2014 Jul 5;14:193. doi: 10.1186/1471-244X-14-193 (PMC4100056; doi:10.1186/1471-244X-14-193)

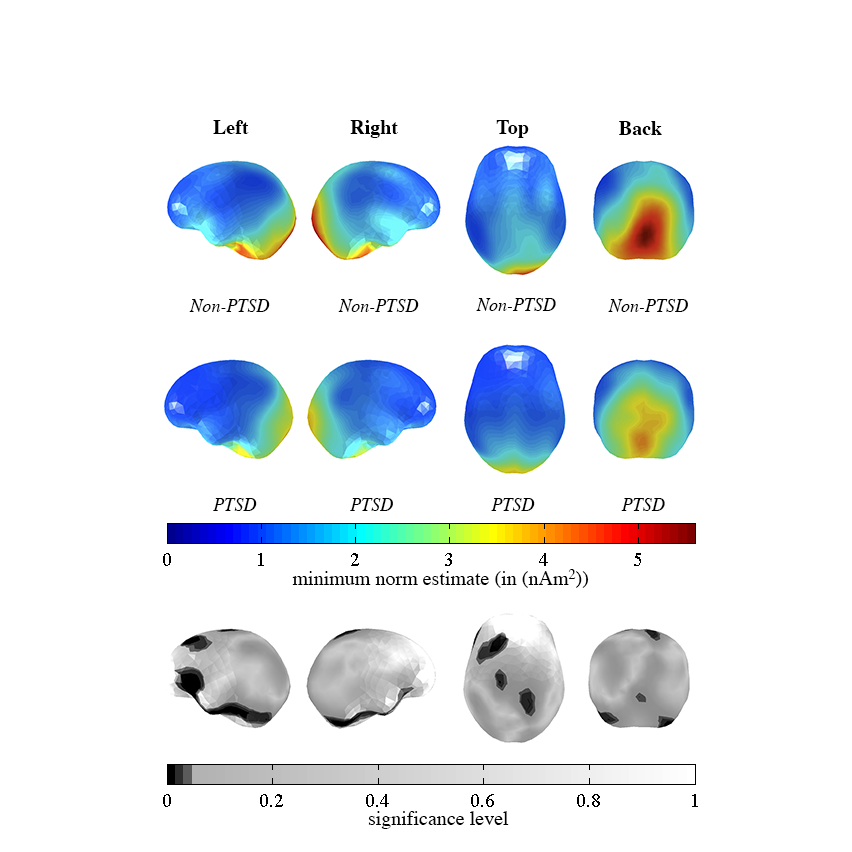

Supplement: Additional file 1 — Brain maps present the minimum-norm estimate (average from 128 to 143 ms) in the unpleasant condition for the Non-PTSD control group and the PTSD group. The brain maps on the lower line show the significance of the group difference between the Non-PTSD control and PTSD group. The brain maps are presented from different perspectives (left, right, top and back view). Posttraumatic Stress Disorder. [file 1471-244X-14-193-S1.tiff]
